# Wikipedia in Vascular Surgery Medical Education: Comparative Study

**DOI:** 10.2196/18076

**Published:** 2020-06-19

**Authors:** Michael Yacob, Shamim Lotfi, Shannon Tang, Prasad Jetty

**Affiliations:** 1 Queen's University Kingston, ON Canada; 2 University of Ottawa Ottawa, ON Canada

**Keywords:** medical education, Wikipedia, vascular surgery, medical student

## Abstract

**Background:**

Medical students commonly refer to Wikipedia as their preferred online resource for medical information. The quality and readability of articles about common vascular disorders on Wikipedia has not been evaluated or compared against a standard textbook of surgery.

**Objective:**

The aims of this study were to (1) compare the quality of Wikipedia articles to that of equivalent chapters in a standard undergraduate medical textbook of surgery, (2) identify any errors of omission in either resource, and (3) compare the readability of both resources using validated ease-of-reading and grade-level tools.

**Methods:**

Using the Medical Council of Canada Objectives for the Qualifying Examination, 8 fundamental topics of vascular surgery were chosen. The articles were found on Wikipedia using Wikipedia’s native search engine. The equivalent chapters were identified in Schwartz Principles of Surgery (ninth edition). Medical learners (n=2) assessed each of the texts on their original platforms to independently evaluate readability, quality, and errors of omission. Readability was evaluated with Flesch Reading Ease scores and 5 grade-level scores (Flesch-Kincaid Grade Level, Gunning Fog Index, Coleman-Liau Index, Simple Measure of Gobbledygook Index, and Automated Readability Index), quality was evaluated using the DISCERN instrument, and errors of omission were evaluated using a standardized scoring system that was designed by the authors.

**Results:**

Flesch Reading Ease scores suggested that Wikipedia (mean 30.5; SD 8.4) was significantly easier to read (*P*=.03) than Schwartz (mean 20.2; SD 9.0). The mean grade level (calculated using all grade-level indices) of the Wikipedia articles (mean 14.2; SD 1.3) was significantly different (*P*=.02) than the mean grade level of Schwartz (mean 15.9; SD 1.4). The quality of the text was also assessed using the DISCERN instrument and suggested that Schwartz (mean 71.4; SD 3.1) had a significantly higher quality (*P*=.002) compared to that of Wikipedia (mean 52.9; SD 11.4). Finally, the Wikipedia error of omission rate (mean 12.5; SD 6.8) was higher than that of Schwartz (mean 21.3; SD 1.9) indicating that there were significantly fewer errors of omission in the surgical textbook (*P*=.008).

**Conclusions:**

Online resources are increasingly easier to access but can vary in quality. Based on this comparison, the authors of this study recommend the use of vascular surgery textbooks as a primary source of learning material because the information within is more consistent in quality and has fewer errors of omission. Wikipedia can be a useful resource for quick reference, particularly because of its ease of reading, but its vascular surgery articles require further development.

## Introduction

Medical education has changed drastically with the increasing use of technology. In particular, internet resources are used by doctors, students, and patients alike to answer clinical questions. Web 2.0 resources such as Wikipedia are rapidly evolving because of their open-source editing community. Currently, there are more than 6 million English articles that are actively monitored and updated by a community of Wikipedia editors [1]. This vast community attracts readers from all backgrounds, from patients seeking medical information to medical professionals needing a quick reference.

In 2009, a survey showed that 80% of physicians use Google, 70% of physicians routinely used Wikipedia, and 53% of physician internet visits involved user-generated web 2.0 resources [2]. Medical students have similarly been observed searching for information online and have identified Wikipedia as a preferred learning resource because of Wikipedia’s ease of access (98% of respondents) and ease of understanding (95% of respondents) [3]. Another study [4] showed that, in addition to university resources, first year medical students used Google and Wikipedia most frequently and rarely accessed recommended journal articles and online textbook chapters.

Generally, Wikipedia has been regarded for its readability, although research [5] has identified a lack of consistency in some subjects. Previous research [6] has found that neurosurgery articles on Wikipedia have worse readability when compared to that of national information articles, and that they do not meet the Centers for Disease Control and Prevention clear communication guidelines for patients. A study [7] observed reading engagement in medical students by tracking their eye movements while reading various online resources and found better engagement with Wikipedia. Another study [8] reported that preclerkship medical students showed improved short-term knowledge acquisition after reading general medical topics on Wikipedia compared to that shown when reading textbooks and UpToDate (an online clinical decision support resource).

However, previous research [5] has also suggested that Wikipedia articles across medical and scientific topics vary in quality. While one study’s [9] findings supported the use of Wikipedia in answering specific questions about pharmacotherapy, another study [10] found gross inaccuracies in articles on topics in otolaryngology when compared with the same topic in a standard textbook of surgery. In recent years, there has been increasing focus on whether it is appropriate for medical students to use web 2.0 resources for learning in specific subspecializations; assessments of cardiology, gastroenterology, and respirology articles have determined that quality and errors of omission are of significant concern when considering Wikipedia as a medical education resource [11-13]. Similar findings were reported when Wikipedia articles were compared to Grant’s Atlas of Anatomy for musculoskeletal anatomy [14].

In this paper, we provide an analysis of the readability and quality of Wikipedia vascular surgery articles by comparing them to a standard surgery textbook. Through this analysis, we hope to better understand whether Wikipedia is suitable as an academic resource for medical students and for junior trainees in the field of vascular surgery.

## Methods

### Identification and Assessment of Content

Common diagnoses in vascular surgery (8 different topics) were identified from the Medical Council of Canada Objectives for the Qualifying Examination [15]. These diagnoses were used as default search terms in Wikipedia’s [16] native search engine; corresponding chapters of the same title were identified from the table of contents of Schwartz Principles of Surgery [17]. Any discrepancies in article identification were resolved by discussion among authors.

### Assessment of Readability

To evaluate the readability of each resource, validated ease-of-reading tools were used. The Flesch Reading Ease score was used to measure reading ease, and the grade levels of each article were determined using 5 different scoring systems—Flesch-Kincaid Grade Level, Gunning Fog Index, Coleman-Liau Index, Simple Measure of Gobbledygook (SMOG) Index, and Automated Readability Index.

The Flesch Reading Ease score [17], represented as a number from 0 to 100, determines the degree of textual difficulty and is calculated as *Flesch Reading Ease score* = 206.835 – (1.015 *ASL*) – (84.5 *ASW*), where *ASL* is the average sentence length and *ASW* is the average number of syllables per word. Scores from 90 to 100 suggest the content is easily understood at a fifth grade level, scores from 80 to 90 suggest the content is easily understood at a sixth grade level, scores from 70 to 80 suggest the content is easily understood at a seventh grade level, scores from 60 to70 suggest the content is easily understood at eighth to ninth grade levels, scores from 50 to 60 suggest the content is easily understood at 10th to 12th grade levels, scores from 30 to 50 suggest the content is easily understood at a college (or university) level, and scores from 0 to 30 suggest the content is at the level of university graduates [6].

Using the Flesch Reading Ease score as a baseline, the other scoring systems were used to determine the education level that the average reader should have in order to understand the text presented. Similarly based on average sentence length and average number of syllables per word, the Flesch-Kincaid Grade Level system [18] calculates a resultant age level that corresponds with respective United States academic grade levels using the equation *Flesch-Kincaid Grade Level* = (0.39 *ASL*) + (11.8 *ASW*) – 15.59. The Gunning-Fog Index calculates grade level using the equation *Gunning-Fog Index* = 0.4 (*ASL* + *PHW*) that considers the average number of words per sentence, *ASL*, and the percentage of hard words, *PHW*. Hard words were considered to be those with 3 or more syllables that were not proper nouns, combinations of easy words, hyphenated words, or two-syllable verbs with -es or -ed endings [6,18]. The Coleman-Liau Index [19] uses the equation *Coleman-Liau Index* = 0.0588 (*average number of letters per 100 words*) – 0.296 (*average number of sentences per 100 words*) – 15.8 that considers the average number of letters and sentences per 100 words. The SMOG Index grade level equation [20] uses the number of polysyllabic words and the total number of sentences:

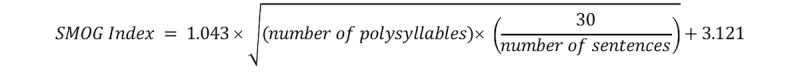



Gunning-Fog Index, Coleman-Liau Index, and SMOG Index scores correspond with American grade levels with scores greater than 13 indicating college (or university) level and above. The Automated Readability Index [18] is calculated using the total number of characters, words, and sentences in the equation *Automated Readability Index* = 4.71 (*characters*/*words*) + 0.5 (*words*/*sentence*) – 21.43 and provides a score from 1 to 14 that corresponds to grade level, where 13 is equivalent to college (or university) level and 14 is at the level of a college (or university) graduate.

These calculations were performed using Office Word software (version 2010; Microsoft Inc) by copying the full text of each article into the word processor and removing all formatting, images, and tables.

### Assessment of Quality

Overall quality and completeness of the resources were assessed with the DISCERN instrument [21] and by identifying errors of omission. To determine the quality of information within, articles were rated on each of the DISCERN instrument’s 16 questions with 5-point responses. DISCERN questions address two key domains and the instrument is typically used to help consumers of health information assess the quality of published information regarding treatment choices. DISCERN questions focus on the reliability of the publication source and whether the information is complete [22]. The final question of the DISCERN instrument considers the reader’s overall impression of quality. For each question, a score of 1 indicated that the resource had serious or extensive shortcomings, a score of 3 indicated some potentially important but not serious shortcomings, and a score of 5 indicated minimal shortcomings. The total score could range from 16 to 80, where 63 to 80 suggested excellent quality, 51 to 62 was good quality, 39 to 50 was fair quality, and 16 to 38 was poor quality [22].

### Assessment of Errors of Omission

Errors of omission were assessed by considering the completeness of the information under categories of epidemiology, pathogenesis, natural history, presenting symptoms, signs on physical examination, noninvasive investigations, invasive investigations, conservative management, medical management, surgical management, endovascular management, and references. Each category, with the exception of references, was assigned a score of 0 if not present, 1 if present but incomplete, or 2 if complete. References were assigned 0 if they were absent or incomplete, or 1 if they were complete. A maximum score of 23 could be assigned to each passage (indicating no errors of omission in any of the aforementioned categories). Errors in each category were counted and totaled per article. To compare between articles, an error of omission rate was calculated by dividing the number of errors by the total word count per article. There was an inverse relationship between the score and the number of errors of omission (or error of omission rate).

### Statistical Analysis

Readability scores were analyzed by taking the mean of the grade level or grade-level equivalent obtained from the Flesch Reading Ease score, Flesch-Kincaid Grade Level, Gunning-Fog Index, Coleman-Liau Index, SMOG Index, and Automated Readability Index. Overall readability of each source (Wikipedia or Schwartz) was calculated by taking the mean of all article scores from that source.

Each passage from Wikipedia and Schwartz Principles of Surgery was independently read and assessed for quality and errors of omission by two junior medical trainees with similar levels of education at the time of the study. The articles were read in their native formats to simulate typical reading circumstances. Mean DISCERN score and mean error of omission rate were calculated for each article by averaging between the two readers prior to further analysis. To ensure the quality of these scores, interobserver concordances (kappa value, κ) were calculated, and any concordance values less than 0.8 were discussed by the research team to clarify the discrepancy. The quality and error of omission rate of each source was determined by taking the mean of all articles from that source.

Readability scores, DISCERN scores, and error of omission rates were compared between corresponding Wikipedia and Schwartz articles using two-tailed independent *t* tests with unequal variances. Statistical significance was defined as *P*<.05. All statistical analyses were performed in Excel (version 2020; Microsoft Inc) using the statistical package add-on.

## Results

### Content Characteristics

Articles on 8 vascular surgery topics were analyzed—carotid artery disease, critical limb ischemia, claudication, acute limb ischemia, aortic dissection, abdominal aortic aneurysm, venous insufficiency, and mesenteric ischemia. At the time of the initial Wikipedia search in July 2013, a search for “carotid artery disease” redirected to an article titled Carotid Artery Stenosis, a search for “critical limb ischemia” redirected to an article titled Peripheral Vascular Disease, and a search for “claudication” redirected to an article titled Intermittent Claudication. The other search terms produced eponymous articles.

An updated search in March 2020, showed that the article titled Peripheral Vascular Disease redirected to an article titled Peripheral Artery Disease, while a search for “critical limb ischemia” redirected to an article titled Chronic Limb Threatening Ischemia. Interestingly, a search for “claudication” resulted in 2 articles—1 entitled Claudication and the other entitled Intermittent Claudication. All other searches resulted in the same pages.

The aforementioned search terms were used to identify subchapters in three vascular surgery–specific chapters of Schwartz Principles of Surgery (ninth edition). The subchapter titled Lower Extremity Arterial Occlusive Disease included Critical Limb Ischemia, Claudication, and Acute Limb Ischemia subheadings. Chronic Venous Insufficiency and Mesenteric Artery Disease subheadings contained the information pertaining to venous insufficiency and mesenteric ischemia, respectively. A comparison with the 11th edition of Schwartz Principles of Surgery yielded no changes.

### Readability Scores

Wikipedia had a mean Flesch Reading Ease score of 30.5 (SD 8.4) across all the articles, while Schwartz had a mean score of 20.2 (SD 9.0) which suggested that Wikipedia articles can be understood by readers at a college (or university) level, while Schwartz content was at the level of a college (or university) graduate.

Using 5 different indices to determine approximate grade level, Wikipedia had a mean grade level of 14.2 (SD 1.3), while the Schwartz had a mean grade level of 15.9 (SD 1.4). These were in agreement with the Flesch Reading Ease scores and placed the Wikipedia articles at a lower grade level than the Schwartz Principles of Surgery text. Both Wikipedia and Schwartz content was for readers at the postsecondary level.

**Figure 1:**
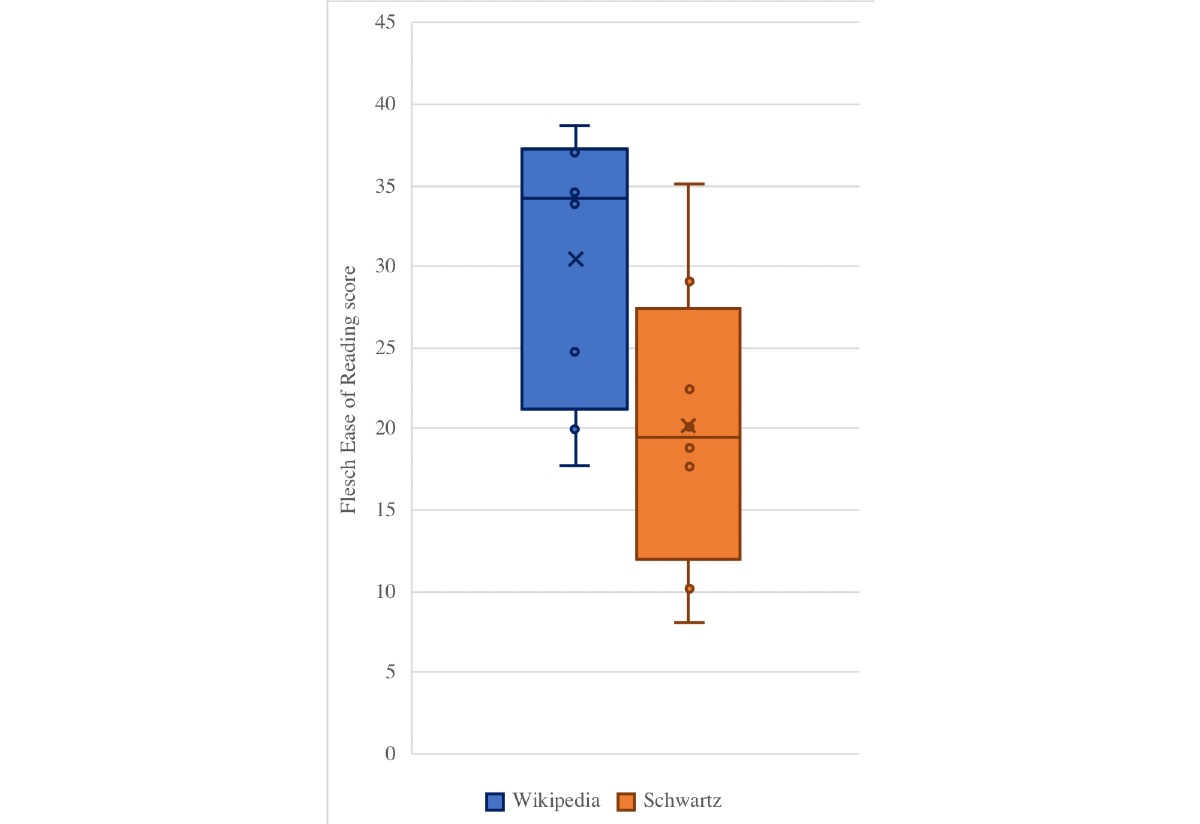
Comparison of Wikipedia and Schwartz average Flesch Reading Ease scores.

**Figure 2:**
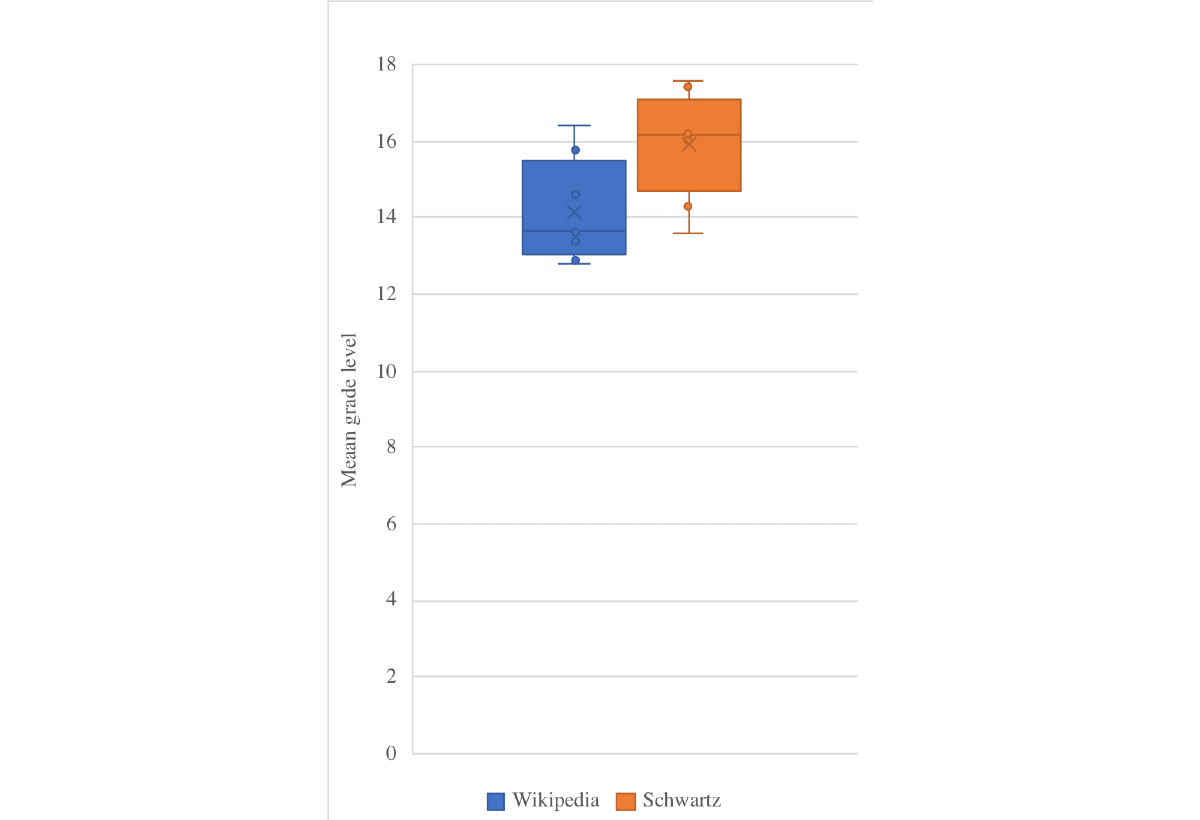
Comparison of mean grade level using Flesch-Kincaid Grade Level, Gunning Fog Index, Coleman-Liau Index, SMOG Index, and Automated Readability Index scores.

The differences between Wikipedia and Schwartz readability scores (Table 1) were statistically significant for Flesch Reading Ease score (*P*=.03), Gunning Fog Index (*P*=.02), Coleman-Liau Index (*P*=.02), SMOG Index (*P*=.04), and Automated Readability Index (*P*=.04), but not for the Flesch-Kincaid Grade Level (*P*=.06). This suggests that the Wikipedia articles were consistently easier to read, and that Schwartz Principles of Surgery was written for a more advanced audience. These relationships are further illustrated in Figures 1 and 2.

**Table 1:** Comparison between Wikipedia and Schwartz Principles of Surgery readability.

Readability assessment	Wikipedia, mean (SD)	Schwartz, mean (SD)	*t* test (*df*)	*P* value
Flesch Reading Ease	30.5 (8.4)	20.2 (9.0)	2.14 (14)	.03
Flesch-Kincaid Grade Level	13.8 (1.6)	15.5 (1.7)	2.14 (14)	.06
Gunning Fog Index	16.6 (1.6)	19.1 (2.0)	2.14 (14)	.02
Coleman-Liau Index	15.0 (1.4)	16.5 (1.0)	2.16 (13)	.02
SMOG Index	12.5 (1.2)	13.9 (1.3)	2.14 (14)	.04
Automated Readability Index	12.9 (1.6)	14.7 (1.4)	2.14 (14)	.04
Mean grade level	14.2 (1.3)	15.9 (1.4)	2.14 (14)	.02

### Quality Assessment

DISCERN scores are shown in Table 2. All of the Schwartz subchapters had scores between 65.0 and 74.5 (ie, all were classified as excellent by DISCERN criteria). In contrast, only the Wikipedia article entitled Peripheral Vascular Disease received a score that was classified as excellent. Of the 8 topics, 4 Wikipedia articles were classified as good, 2 were classified as fair, and 1 was classified as poor according to their DISCERN score. On the whole, Schwartz Principles of Surgery subchapters (mean 71.4; SD 3.1) performed significantly better than Wikipedia (mean 52.9; SD 11.4) on the DISCERN scoring criteria (*P=*.002). This suggests that the content of the text from Schwartz Principles of Surgery is superior in quality to that of the text from Wikipedia.

Interobserver concordance ranged from κ=0.68 to κ=0.96 for Wikipedia and from κ=0.64 to κ=1.00 for Schwartz. Interobserver concordance values less than 0.8 were observed for the Wikipedia article on aortic dissection and the Schwartz subchapters on acute limb ischemia and aortic dissection; however, the overall final scores were similar and were thus deemed acceptable.

**Table 2:** Comparison of Wikipedia and Schwartz Principles of Surgery DISCERN scores indicating content quality.

Article topics	DISCERN, mean of both readers	
	Wikipedia	Schwartz
Carotid artery disease	62.0	74.0
Critical limb ischemia	64.5	73.0
Claudication	45.0	73.5
Acute limb ischemia	56.5	71.0
Aortic dissection	57.5	70.0
Abdominal aortic aneurysm	61.0	74.5
Venous insufficiency	31.0	70.5
Mesenteric ischemia	45.5	65.0

### Errors of Omission

The total number of errors of omission for each article are demonstrated in Table 3. Overall, Wikipedia articles contained a significantly greater number of errors of omission (*P=*.008) compared to Schwartz Principles of Surgery. Notably, the Wikipedia article on critical limb ischemia (Peripheral Vascular Disease) was extremely incomplete with a score of 1 out of a maximum of 23. The mean errors of omission scores for Wikipedia was 12.5 (SD 6.8)).The highest scoring article on Wikipedia was on the topic of abdominal aortic aneurysm with a score of 21.5 points. In contrast, most articles for Schwartz Principles of Surgery scored high with a mean of 21.3 (SD 1.9) points, and the subchapter on chronic venous insufficiency scored the lowest at 17 points. From these results, we can reasonably infer that Schwartz Principles of Surgery is a more complete resource compared to Wikipedia for vascular surgery topics.

Interobserver concordance for the Wikipedia assessments ranged from κ=0.77 to κ=1.00, while interobserver concordance for Schwartz Principles of Surgery ranged from κ=0.08 to κ=1.00 (Table 4). The subchapter of Venous Insufficiency from Schwartz had an interobserver concordance of κ=0.08 which reflected a number of incomplete sections in the text; however, the final errors of omission scores were ultimately similar between raters, so the mean score was nevertheless used in the statistical analysis. Further scoring breakdowns are available in Multimedia Appendix 1.

**Table 3:** Comparison of Wikipedia and Schwartz Principles of Surgery errors of omission.

Article topics	Errors of omission, mean of both readers	
	Wikipedia	Schwartz
Carotid artery disease	12.0	22.0
Critical limb ischemia	1.0	23.0
Claudication	9.0	22.0
Acute limb ischemia	14.5	23.0
Aortic dissection	19.5	21.0
Abdominal aortic aneurysm	21.5	20.5
Venous insufficiency	6.5	17.0
Mesenteric ischemia	16.0	21.5

**Table 4:** Interobserver concordance values for DISCERN and error of omission assessments.

Article topics	DISCERN interobserver concordance		Errors of omission interobserver concordance	
	Wikipedia	Schwartz	Wikipedia	Schwartz
Carotid artery disease	0.90	1.00	0.92	1.00
Critical limb ischemia	0.94	1.00	1.00	1.00
Claudication	0.84	0.86	0.82	1.00
Acute limb ischemia	0.84	0.64	0.90	1.00
Aortic dissection	0.68	0.66	0.81	0.36
Abdominal aortic aneurysm	0.82	0.82	0.77	0.67
Venous insufficiency	0.96	1.00	0.97	0.08
Mesenteric ischemia	0.96	1.00	0.90	0.77

## Discussion

Common vascular surgery topics (8 topics) were selected from the Medical Council of Canada Objectives for the Qualifying Examination, and their equivalent articles were identified on Wikipedia and in subchapters in Schwartz Principles of Surgery. Through analysis of the readability and quality of the content of these sources, we investigated the suitability of Wikipedia as a vascular surgery resource for medical students.

We found that the quality of Wikipedia articles was mostly classified as good or fair by DISCERN criteria (Table 2), and that Wikipedia articles were written at the college (or university) level (Figure 1). There were numerous errors of omission in many of the Wikipedia articles, and some articles did not contain fundamental subsections (Table 3). Schwartz Principles of Surgery was found to contain consistently higher quality content (classified as excellent by DISCERN criteria) and contained fewer errors of omission (Tables 2 and 3) but was also found to have lower readability (Figure 1). This was attributed to the length explanations of concepts in the text which contained additional supporting figures and tables and likely contributes to the higher grade level that was associated with the text.

While the Wikipedia articles were found to have a lower grade level and higher reading ease compared to that of Schwartz (Table 1 and Figure 2), both sources still required that the reader be comfortable with reading at the college (or university) level. The difference in reading level required for Wikipedia articles compared to that required of textbook passages, as well as Wikipedia’s ease of access likely is the reason it and other online resources are appealing to users [2-4].

Existing research [7] suggested that preclerkship medical students performed better on short-term knowledge acquisition tests after studying from Wikipedia compared to when they studied from a textbook. This could be as a result of the presence of superfluous background information in the textbook, and the fact that Wikipedia is written for the general population. Because of their general target audience, Wikipedia can be written in such a way that key points are summarized simply without the need for extensive explanation which allows for faster knowledge acquisition, especially for newer medical students.

It is important to consider that Scaffidi et al’s study [7] on Wikipedia content and quality focused on general medical topics, whereas other studies [11-13] have focused on specialized medical fields such as cardiology, respirology, and gastroenterology and have found findings contradictory to those of Scaffidi et al. Another study [14] focusing on musculoskeletal anatomy content on Wikipedia discovered inaccuracies and errors of omission as well as reporting that many references were not appropriate. In fact, these studies [11-14] do not recommend Wikipedia as a learning resource for medical students because of the significant errors of omission and low-quality content.

In vascular surgery topics, many Wikipedia articles were incomplete, and the many inconsistencies that were noted on the website that continue to be unresolved at the time this paper was written. An example of an inconsistency observed on Wikipedia is the presence of two separate articles titled Claudication and Intermittent Claudication, both describing the condition associated with vascular claudication in peripheral artery disease [23,24]. In addition, there are a number of statements that lack appropriate references (for example, in the article titled Claudication, numerous statements are note-cited). Citations on Wikipedia are recommended to be from reliable secondary or tertiary resources, and this is not true in a number of the vascular surgery articles [25].

The open-source nature of Wikipedia and other web 2.0 resources is one of its greatest strengths, but it can also be one of its greatest weaknesses. Wikipedia is a vast resource with over 100,000 actively contributing editors from around the world [26]. It is moderated by these editors who also strive to maintain the quality of the resource; however, in a specialized field such as vascular surgery there may be difficulty in validating content accuracy due a dearth of available or knowledgeable editors. In this era of rapid information turnover, it could be argued that textbooks cannot be updated as quickly as an open-source website such as Wikipedia. This ability to be kept up-to-date in combination with its ease of access makes Wikipedia a valuable resource for medical information and is the reason it is widely used by the general population and medical professionals alike [2-4].

In this study, we critically examined the quality, completeness, and readability of Wikipedia articles on common vascular surgery topics and compared them to corresponding excerpts from Schwartz Principles of Surgery. This study used validated tools, such as the Flesch Reading Ease scoring system and the DISCERN instrument. Five readability indices were used to minimize bias when calculating grade level and similar results were obtained which supports their validity. The DISCERN instrument and error of omission assessment introduce some subjectivity into the study as a result of individual interpretation. In addition, the items on the errors of omission rating scale were developed based on standard presentation in medical education and requires further assessment to demonstrate its validity. Presentation of information in textbooks and Wikipedia may vary between the sources for better readability in that particular source. For example, information regarding epidemiology may be combined with natural history to aid the reader in connecting these concepts. These variations were often the reason for the low interobserver concordance values in Table 4.

Ultimately, the use of Wikipedia in medical education should not be disregarded. It has the potential to serve as a powerful reference for all users, but medical professionals and students should be aware that articles on Wikipedia are written with the general population in mind. This study demonstrates that further development is required for vascular surgery topics on Wikipedia before it can be reliably recommended as a resource for medical trainees. Currently, surgical textbooks are more likely to reflect the depth and breadth of knowledge required for medical learners in the field of vascular surgery. Further research could examine how web 2.0 resources are utilized depending on the level of the trainee, and the motivations for choosing a particular resource at a particular stage of clinical knowledge acquisition.

## Appendix

Multimedia Appendix 1 Raw data set and statistical analysis details.

## Abbreviations

SMOG: Simple Measure of Gobbledygook index
